# Navigating the AI Frontier in Toxicology: Trends, Trust, and Transformation

**DOI:** 10.1007/s40572-025-00514-6

**Published:** 2025-12-05

**Authors:** Thomas Luechtefeld, Thomas Hartung

**Affiliations:** 1 ToxTrack LLC, Bethesda, MD 20894 USA; 2 Insilica Inc., Rockville, MD 20848 USA; 3 Center for Alternatives to Animal Testing (CAAT), Baltimore, MD 21205 USA; 4 Doerenkamp-Zbinden-Chair for Evidence-Based Toxicology, Johns Hopkins Bloomberg School of Public Health, 615 N Wolfe St, Baltimore, MD W703221201 USA; 5 CAAT-Europe, University of Konstanz, Konstanz, 78464 Germany; 6 CAATevents, Solingen, 42651 Germany

**Keywords:** Artificial Intelligence (AI), Toxicology, New Approach Methodologies (NAM), e-Validation, Explainable AI (xAI), Chemical risk assessment, Regulatory science, Bias audit, Digital twins, Causal modeling, Responsible AI, Human relevance, TREAT principles, Ethical toxicology, Federated learning

## Abstract

**Purpose of Review:**

The integration of artificial intelligence (AI) into toxicology marks a profound paradigm shift in chemical safety science. No longer limited to automating traditional workflows, AI is redefining how we assess risk, interpret complex biological data, and inform regulatory decision-making. This article explores the convergence of AI and other new approach methodologies (NAMs), emphasizing key trends such as multimodal learning, causal inference, explainable AI (xAI), generative modeling, and federated learning.

**Recent Findings:**

These technologies enable more human-relevant, mechanistically grounded, and ethically aligned toxicological predictions—surpassing the reproducibility and scalability of animal-based methods. However, the dynamic nature of AI models challenges traditional validation paradigms. To address this, we introduced the e-validation framework, which operationalizes the TREAT principles (Trustworthiness, Reproducibility, Explainability, Applicability, Transparency) and incorporates AI-powered modules for reference chemical selection, virtual study simulation, mechanistic cross-validation, and post-validation surveillance through companion agents. Ethical considerations—including bias audits, equity audits, and participatory governance—are also foregrounded as critical elements for responsible AI adoption. The emergence of a co-pilot model, where AI augments but does not replace human judgment, offers a pragmatic path forward. Supported by evidence from the 2025 Stanford AI Index and recent regulatory advances, we argue that the infrastructure, economics, and policy momentum are now aligned for global-scale deployment of AI-based toxicology.

**Summary:**

The future of the field lies not in replicating legacy practices, but in reinventing toxicology as an adaptive, transparent, and ethically grounded science that delivers more accurate, inclusive, and human-centric safety assessments.

**Lay Summary:**

Artificial intelligence (AI) is changing how we test chemicals for safety. Instead of using animals, new computer-based tools can predict how substances affect human health more quickly, accurately, and ethically. This article looks at how these technologies—like smart data systems, models that explain their reasoning, and even AI "agents" that run simulations—can improve toxicology. We also introduce a new idea called "e-validation", which uses AI to help validate these methods in real-time, not just once. This ensures the models stay up to date and reliable. But using AI safely means tackling big questions: Can we trust results we don't fully understand? How do we prevent unfairness or bias in the data? We suggest a "co-pilot" model, where AI supports, but doesn't replace, human experts. With better data sharing, strong ethics, and smarter oversight, AI can help make chemical safety testing more human-focused, fair, and effective.

## Introduction: A Paradigm Shift

The integration of artificial intelligence (AI) into toxicology is no longer a speculative vision [1]; it is a scientific necessity. Over the past two decades, toxicology has evolved from a primarily observational discipline of black-box animal models and some human data to a data-rich science poised for algorithmic innovation. Fueled by the proliferation of big data from high-throughput screening, ~ omics, digital pathology, and curated chemical databases as well as machine-readable scientific literature, the field has entered a new era where AI is not merely a tool—it is a transformative force. This shift is underpinned by the convergence of three accelerating forces: the exponential growth of toxicological data, unprecedented gains in computational power, and rapid advances in machine learning algorithms [2]. Together, they are reshaping how we interrogate toxicity, predict risk, extract mechanistic insight, and ultimately make regulatory decisions. In this context, AI offers more than automation—it represents a redefinition of toxicological practice.

Modern toxicology must now contend with the "Five Vs" of big data—volume, velocity, variety, veracity, and value [3]. These dimensions collectively exceed the capacity of traditional methods and demand scalable, adaptive computational approaches. AI excels at distilling signal from complexity, enabling predictive modeling across structurally diverse chemicals, integration of heterogeneous evidence streams, and synthesis of multi-omics and legacy data.

At the forefront of this revolution are AI-based New Approach Methodologies (NAMs), which promise not only greater speed and cost efficiency, but also enhanced human relevance and ethical acceptability. Tools such as deep neural networks, graph-based learning, natural language processing, and generative models are enabling new paradigms—from automated read-across and mechanistic hypothesis generation to probabilistic risk assessment and evidence synthesis [4]. These emerging paradigms are being benchmarked through comparative validation studies. For instance, RASAR models achieved 87% balanced accuracy across nine OECD guideline endpoints—surpassing the ~ 81% reproducibility of the respective animal studies [5]. Probabilistic read-across and causal-inference models similarly demonstrate enhanced mechanistic resolution and reproducibility over traditional toxicity tests. Such metrics provide quantitative evidence that AI-based NAMs outperform legacy paradigms while improving interpretability and consistency.

However, this transformation is not without challenges. AI systems, particularly deep learning models, often suffer from interpretability issues, raising concerns about trust and transparency—core requirements in regulatory toxicology. Furthermore, biases in training data, lack of standardization, and gaps in cross-disciplinary expertise impede broader adoption. As emphasized in the TREAT framework [6] and e-validation paradigm [7], rigorous validation, explainability, and human oversight must be embedded throughout the model lifecycle.

The maturation of AI-readiness in toxicology is evident: inference costs have dropped by over 280-fold in just two years, performance saturation is driving the shift from generic to domain-specific models, and regulatory precedents are being set, as seen in the FDA's approval of over 200 AI-enabled devices in 2023 [7]. Toxicology must now seize this momentum to define how AI can be responsibly, ethically, and scientifically integrated into every stage of chemical safety assessment. In short, toxicology stands at an inflection point. The discipline must evolve from merely adopting AI to actively co-developing it—ensuring that the next generation of safety science is not only data-driven, but also mechanistically grounded, socially responsible, and globally harmonized.

Equally central to responsible innovation is the social dimension. Transitioning toward AI-enabled toxicology will not displace the scientific workforce but rather redirect expertise. Routine in-vivo assay technicians can transition toward data-curation, validation, and quality-assurance roles; regulatory scientists can specialize in AI oversight, bias auditing, and ethical governance. Investment in up-skilling and continuing education must therefore accompany technical deployment to ensure workforce sustainability and social responsibility.

## The Emerging AI Landscape: What's New in 2025?

**Fig. 1:**
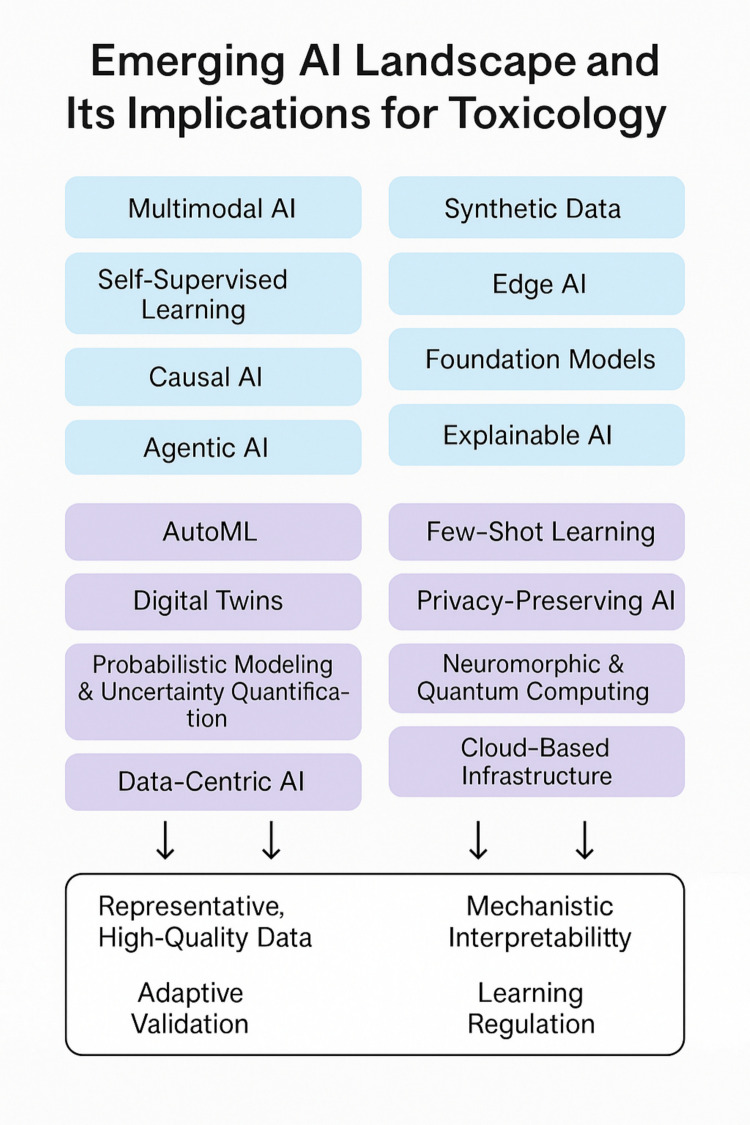
**The Emerging AI Landscape and its Implications for Toxicology**. A number of trends in AI technological developments were identified and their impact on toxicology is summarized. Boxes in blue indicate current trends, those in purple the longer-term impactors. The white box summarizes the expected impacts on the field

In Hartung & Kleinstreuer (2025, Table 1 therein) [6] discussing AI-validation, we highlighted key AI trends poised to reshape toxicology. These include (Fig. 1):

### Foundations: Data, Modeling, and Infrastructure

*Data-Centric AI and FAIRification*—In contrast to model-centric AI approaches that focus on algorithmic sophistication, data-centric AI prioritizes dataset quality, curation, and infrastructure. This paradigm is particularly relevant to toxicology, where data heterogeneity, legacy formats, and annotation gaps often undermine model performance. Initiatives such as the EPA's CompTox Chemistry Dashboard,^1^ OECD's QSAR Toolbox,^2^ and NIEHS's Integrated Chemical Environment (ICE)^3^ exemplify data FAIRification—making data findable, accessible, interoperable, and reusable.^4^ Reusable FAIR Implementation Profiles [8] appear to be a way forward here [9]. AI systems built on curated, standardized, and semantically annotated data are not only more accurate but also more trustworthy and transparent. Moreover, data-centric workflows increasingly incorporate AI tools themselves to assist in cleaning, labeling, harmonizing, and flagging inconsistencies in toxicological datasets. These "AI-curates-AI" cycles are essential for scaling up robust, reproducible, and generalizable toxicology models across institutions, jurisdictions, and species.^5^ The old mantra of *trash in, trash out* still holds, but it is no longer the humans sorting the trash but the machines.

Equity-aware data governance is critical. Scenarios in which low-quality or biased ‘trash data’ disproportionately represent specific ethnic or geographic sub-populations can perpetuate inequities. FAIRification efforts should therefore include demographic representativeness audits, targeted high-quality data collection where gaps exist, and alignment with CARE (Collective Benefit, Authority to Control, Responsibility, and Ethics) principles to ensure inclusive datasets.

*Self-supervised learning (SSL)* offers a transformative advantage for toxicology, where data scarcity, label noise, and high annotation costs often limit the applicability of supervised machine learning. SSL leverages unlabeled data—abundant in toxicological literature, chemical registries, omics repositories, and legacy datasets—to learn useful data representations without requiring predefined labels. This pretraining process allows downstream tasks, such as toxicity prediction or chemical classification, to be fine-tuned on smaller, high-quality labeled datasets, dramatically enhancing model performance and generalizability. For instance, SSL techniques can extract embeddings from chemical graphs or transcriptomic time-series, which can then be used to cluster compounds, identify outliers, or predict missing annotations. Given the abundance of historical toxicology data that are incomplete or inconsistently labeled, SSL could unlock substantial latent value by turning raw, unlabeled information into a source of predictive insight [10–12].

*Synthetic data* generation—via methods such as generative adversarial networks (GANs), variational autoencoders, or large language models—is increasingly viewed as a practical solution to toxicology's chronic data scarcity and privacy constraints. Synthetic datasets can be used to augment training sets, simulate underrepresented scenarios, or generate in silico populations for probabilistic risk assessment. This approach has been particularly useful in drug discovery and genomics, where data sharing restrictions or rare events limit model training. However, the toxicological utility of synthetic data remains debated, particularly in terms of biological plausibility and statistical fidelity. Overfitting to synthetic artifacts, loss of real-world variance, and "data nutritional deficiency" are valid concerns. As such, rigorous quality assessment of synthetic datasets is required before they can be trusted for regulatory decision-making. Nonetheless, when carefully designed and benchmarked, synthetic data may become a valuable complement to traditional datasets—particularly in precompetitive or federated toxicology initiatives [13, 14].

*Foundation models*—large-scale, pre-trained models developed on broad datasets— have become the backbone of modern AI. By pre-training on massive text, image, or biological sequence corpora, models such as GPT-4 (OpenAI, 2023) [15], Med-PaLM [16], and AlphaFold [17] capture rich general-purpose representations that can be fine-tuned or prompt-tuned for toxicology tasks—literature mining for adverse outcome pathways, graph-based chemical classification, or proteomic interaction prediction—without the resource intensity of training from scratch. However, their scale and opacity raise valid concerns about transparency, reproducibility, and regulatory suitability. To address this, the Foundation Model Transparency Index^6^ provides a 100-indicator framework assessing model providers’ disclosure practices,^7^ while the Explainable AI Toolkit (XAITK)^8^ offers modular xAI components (e.g., saliency maps [18], SHAP values, counterfactual explanations) that can be integrated into toxicology-focused pipelines to illuminate decision logic and satisfy interpretability requirements [19].

**Fig. 2:**
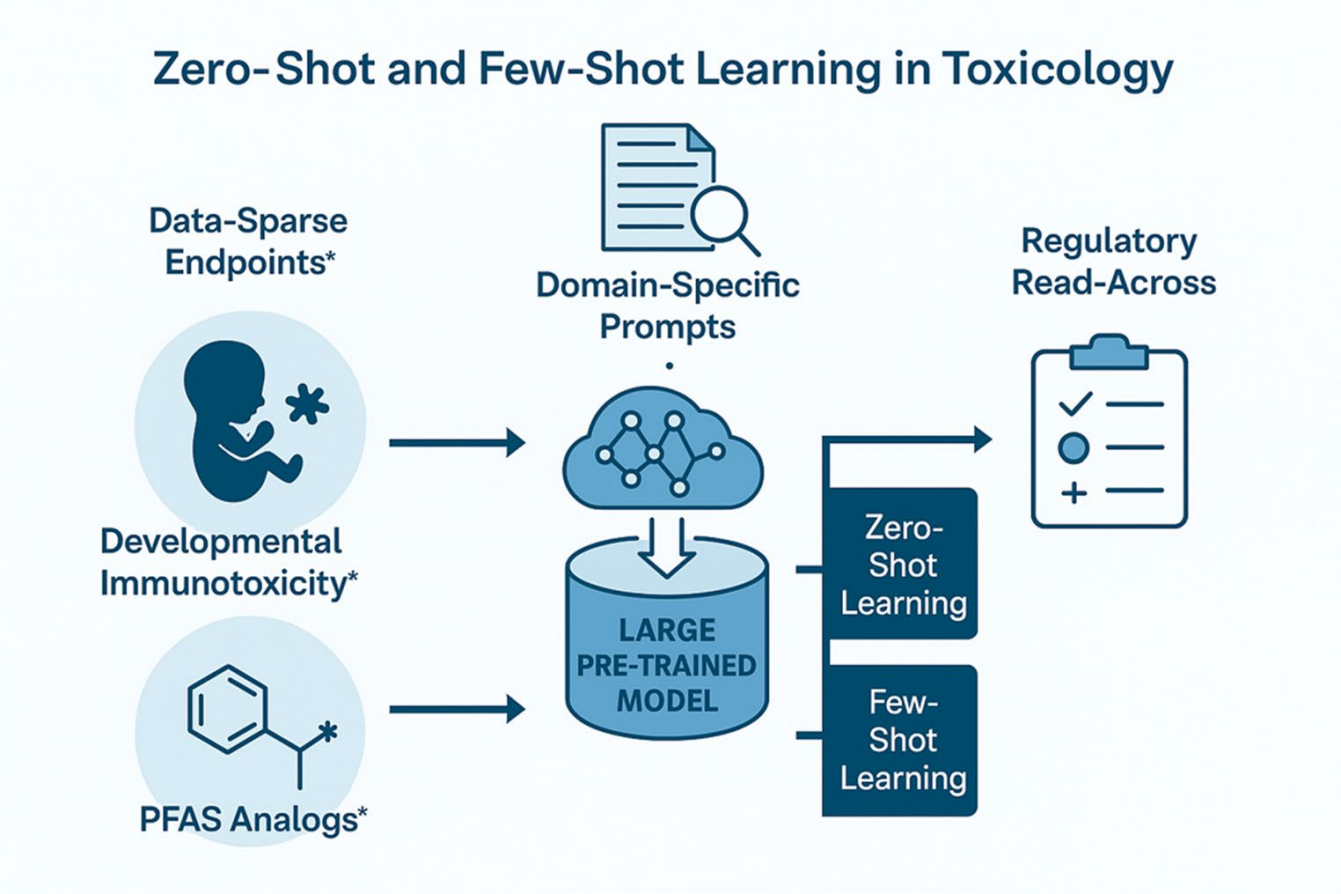
**Zero- and few-shot learning for toxicology workflows**. The graphic is visualizing how zero- and few-shot learning plug into toxicology workflows (e.g., data-sparse endpoints to regulatory read-across); Developmental Immunotoxicity and per- and polyfluoroalkyl substances (PFAS) are used as examples of being sparse for (good) data

*Zero-shot and few-shot learning* methods represent a frontier for applying AI in underexplored areas of toxicology. Unlike traditional machine learning models, which require large, annotated datasets, zero-shot learning allows models to generalize to new toxicity endpoints or chemical classes without prior exposure to annotated examples (Fig. 2). This is achieved by leveraging semantic relationships, often via embeddings or prompt-based learning in large pre-trained models, such as foundation models like GPT-4 or Med-PaLM.^9^ Few-shot learning, in contrast, allows for rapid model adaptation using only a small number of labeled instances, making it ideal for toxicological domains with limited training sets, such as developmental immunotoxicity or endocrine disruption [20]. These methods are particularly advantageous when assessing rare endpoints or substances, where high-quality labeled data is limited or difficult to generate. When coupled with domain-specific prompts or ontologies, large language or multimodal models can infer hazard potential, enabling applications like automated chemical grouping, virtual screening, and read-across with minimal empirical input [2]. Such techniques significantly enhance the scalability and agility of AI systems in (non-)regulatory toxicology (Fig. 2), offering a path forward for predictive toxicology that is both efficient and aligned with human-relevant outcomes. Prominent examples are regulatory read-across and grouping [21, 22] and “benign-by-design”, in the EU called “safe and sustainable by design” (SSbD), framework of Green Toxicology [23, 24], i.e., the toxicological aspects of Green Chemistry avoiding toxic liabilities early in the product development process.

*Parallel and Cloud-Based Compute Infrastructure*—The integration of scalable compute resources—enabled by cloud services and parallel processing architectures—has lowered the entry barrier for toxicological AI. Cloud platforms like AWS, Azure, and Google Cloud allow rapid scaling of storage and compute power, enabling large-scale training on toxicological datasets or batch evaluations of QSAR models. Parallelization frameworks (e.g., Spark,^10^ Ray,^11^ or Dask^12^) can accelerate high-throughput workflows, such as large-scale screening of virtual compound libraries or Monte Carlo simulations in probabilistic risk assessment. Importantly, this infrastructure supports collaborative toxicology through centralized data lakes, version control, and reproducible pipelines. By enabling real-time analysis, cross-institutional modeling, and continuous integration of new data, these tools facilitate a dynamic, open innovation ecosystem that advances both predictive capacity and transparency in toxicological research [25].

### Modeling the Complexity of Toxicological Phenomena

*Multimodal AI* represents one of the most powerful evolutions in toxicological modeling by enabling the integration of diverse data types—including text, ~ omics (genomics, transcriptomics, metabolomics), chemical structures, and imaging data—into a single analytical framework. In toxicology, this approach is particularly advantageous given the multifactorial nature of toxicity mechanisms, which often require correlating molecular-level perturbations with phenotypic or pathological endpoints. Applications of multimodal AI include digital pathology for objective tissue assessment, multi-omics integration to elucidate toxicity pathways, and real-time exposure monitoring through the fusion of environmental sensor data with biological responses. These capabilities significantly enhance the translational potential of AI-driven predictions, bridging the gap between high-content mechanistic data and regulatory endpoints. As toxicology transitions from apical observations to pathway-based assessments, multimodal architectures are poised to become the analytic backbone of next-generation safety science [26].

*Causal AI* marks a fundamental shift in the epistemological foundation of computational toxicology by moving beyond association-based models toward causation-based inference. Traditional AI models excel at identifying statistical correlations but often fall short when tasked with uncovering mechanistic pathways or predicting the consequences of perturbing biological systems. Causal inference frameworks—especially those grounded in directed acyclic graphs, structural equation models, or counterfactual analysis—enable the identification of key drivers within adverse outcome pathways (AOPs) [27], facilitating mechanistic validation and regulatory acceptance. LLM are increasingly capable of supporting mechanistic reasoning or for example the fulfillment of Bradford-Hill criteria for causality [28]. These tools allow toxicologists to simulate "what-if" scenarios, predict the effects of interventions, and prioritize molecular initiating events based on causal relevance rather than statistical association alone. Such capabilities are essential for risk assessment, hazard identification, and the development of human-relevant NAMs. As data-rich toxicology continues to mature, causal AI may redefine how safety science understands and quantifies chemical effects on biological systems [29].

*Physics-informed AI* (PI-AI), and actually more science-informed AI, combines data-driven learning with established mechanistic models—such as toxicokinetics, cell signaling, or biotransformation dynamics—to improve generalizability and credibility of predictions. These hybrid models incorporate differential equations or physical constraints into the AI learning process, ensuring that outputs remain scientifically plausible and interpretable. In toxicology, PI-AI could improve IVIVE (in-vitro-to-in-vivo-extrapolations) predictions [30], PBPK model accuracy, or dose–response extrapolations by embedding known pharmacodynamic principles. For regulators, the integration of first-principles modeling offers reassurance that AI systems are not just statistical correlations, but grounded in biological realism. This synthesis of mechanistic and statistical modeling represents a critical step toward harmonizing AI outputs with regulatory frameworks and translational toxicology needs [31].

*Probabilistic Modeling and Uncertainty Quantification* enables toxicological risk assessments to move beyond binary hazard classification toward nuanced, uncertainty-aware decision-making. AI models—especially Bayesian neural networks and ensemble learning systems—can provide confidence intervals and probabilistic risk estimates that account for biological variability, data gaps, and extrapolation uncertainty. These models support more robust safety margins and better-informed regulatory decisions, particularly for chemicals with sparse in vivo data. Advanced techniques in uncertainty quantification, such as Monte Carlo dropout,^13^ deep ensembles,^14^ or variational inference,^15^ are waiting to be implemented in toxicology to identify influential parameters and prioritize data acquisition. Furthermore, probabilistic read-across and risk assessment (ProbRA) [20, 32, 33] models are being developed as a refinement of conventional structural analog approaches, incorporating prior knowledge and real-world exposure variance. These frameworks support a more human-centric and evidence-based toxicology [34]—critical for translating AI insights into risk governance.

### Democratizing Access and Building Adaptability

*Explainable AI (xAI)*—Explainability is not merely a desirable feature for AI in toxicology—it is a prerequisite for regulatory trust, scientific rigor, and ethical accountability. While deep learning models may offer superior predictive performance, their "black-box" nature limits adoption in high-stakes domains like chemical safety assessment. Explainable AI (xAI) addresses this limitation by providing interpretive insights into model behavior, enabling users to understand why a prediction was made and what features contributed most to it. Techniques such as SHAP values,^16^ i.e., a game-theoretic approach used to explain the output of any machine learning model, local interpretable model-agnostic explanations (LIME) [35], i.e., a method that explains the predictions of any machine learning model by approximating it locally with a simpler, interpretable model, as well as integrated gradients, and rule extraction methods can be used to illuminate complex model outputs.^17^ xAI also facilitates alignment with mechanistic toxicology frameworks, such as adverse outcome pathways (AOPs), by linking predictions to plausible biological mechanisms. As emphasized by both DARPA and the OECD, explainability is increasingly viewed not just as a technical feature but as a governance requirement for the use of AI in toxicological risk assessment [36].

*AutoML and No-Code AI*—The rise of automated machine learning (AutoML) and no-code AI platforms democratizes (toxicological) modeling by lowering the barrier to entry. AutoML frameworks such as Google's Vertex AI,^18^ H2O.ai,^19^ or AutoSklearn^20^ allow users to build, optimize, and validate machine learning pipelines with minimal manual intervention. For toxicology, this means domain experts without formal training in data science can rapidly develop predictive models for specific endpoints, such as hepatotoxicity or endocrine disruption. These tools automate tasks like feature selection, hyperparameter tuning, and performance benchmarking, freeing up resources and accelerating hypothesis generation. No-code platforms further can extend accessibility by offering drag-and-drop interfaces that support toxicological workflows from dataset ingestion to result visualization. While these tools promise to promote inclusivity and speed, they must be coupled with best practices in validation [7, 37], reproducibility, and model documentation to ensure scientific integrity and regulatory alignment.

*Adaptive and continual learning* systems enable AI models to evolve with new data—critical in a field like toxicology, where understanding of chemical mechanisms and regulations is constantly expanding: PubMed alone gives about 13,000 articles per year with the term”toxicology” over the last decade. These systems allow AI models to incrementally update their knowledge base, retrain with emerging evidence, and adapt to shifts in data distribution, such as new formulations or exposure routes. Companion AI agents for post-validation monitoring, as proposed in your e-validation framework [7], exemplify this trend by tracking model performance over time and signaling when retraining or recalibration is needed. Adaptive AI systems are also crucial for maintaining consistency in toxicological assessments as new AOPs are developed or reference standards change. These dynamic capabilities make continual learning indispensable for trustworthy, up-to-date safety decision support systems.

### Scaling Collaboration and Privacy-Preserving Analysis

*Edge AI*, also known as on-device AI, and federated learning provide enabling architectures for decentralized toxicological modeling and privacy-preserving analysis. In scenarios where data cannot be centrally pooled—such as multi-center studies, proprietary industrial datasets, or human subject data—federated learning allows model training across distributed nodes without data transfer. Edge AI further extends this capability by enabling real-time toxicological inference at the site of data generation, such as sensors in environmental monitoring stations or wearable biosensors. These approaches reduce latency, enhance privacy, and facilitate context-aware modeling in situ. For instance, federated toxicology could harmonize predictive models across pharmaceutical partners without exposing proprietary compounds, while edge AI could power on-device exposure assessments in occupational health settings. These technologies align well with ethical data stewardship, especially under General Data Protection Regulation (GDPR) law of the European Union^21^ and related frameworks, and offer scalable pathways toward global, collaborative toxicology without compromising privacy or security.

However, decentralized computation introduces new disparities. To maintain result consistency across low- and high-resource nodes, federated frameworks should incorporate standardized validation toolkits, model cards, and inter-node proficiency testing. ISO 42001^22^ and OECD AI^23^ Principles provide templates for harmonizing quality control and ethical oversight in such distributed environments.

*Privacy-preserving AI* technologies—such as homomorphic encryption, i.e., a type of encryption that allows computations to be performed on encrypted data without decrypting it first, secure multi-party computation (SMPC),^24^ i.e., a cryptographic technique that allows multiple parties to collaboratively compute a function on their private inputs while keeping those inputs secret, and differential privacy, i.e., a technique that allows for data analysis and sharing while protecting the privacy of individual data points protects user data from being traced back to individual users—are increasingly critical in toxicology, particularly when handling sensitive human exposure or biomonitoring data. These techniques enable the training and deployment of AI models without direct access to raw data, thereby safeguarding propriety and confidentiality while still extracting value. Beyond de-identification, ethical deployment requires explicit consent for algorithmic data use. Dynamic consent models—allowing participants to grant, withdraw, or refine permission for AI-based analysis—should be embedded in toxicology data-collection workflows. Consent forms should transparently specify potential secondary uses of anonymized data by AI systems. In collaborative toxicological research or regulatory decision-making, where industry hesitancy over intellectual property and patient privacy concerns can limit data sharing, these approaches facilitate secure collaboration. Privacy-preserving AI also supports compliance with frameworks like the GDPR law of the European Union 25 or the US Health Insurance Portability and Accountability Act (HIPAA),^26^ making it an essential component of trustworthy AI ecosystems. When combined with federated learning, these technologies could create secure, distributed toxicology infrastructures that transcend institutional and jurisdictional barriers.

### Advanced Horizons: Automation and New Hardware

*Agentic AI*, or AI agents capable of autonomous reasoning and task execution, introduces the potential for self-directed workflows in toxicology—from literature review and study design to experimental optimization and data interpretation. These agents, built upon large language models and reinforcement learning frameworks, can plan, execute, and revise multistep procedures, offering a vision of fully automated toxicological pipelines. This is probably the most transformative part of current developments, as it changes the role of AI from a knowledge acquiring and digesting algorithm on command to an orchestrator of active inquiry and self-optimization. For instance, an AI agent might design a virtual screening experiment, adjust dose ranges based on early feedback, and generate preliminary risk assessments. However, the practical deployment of such systems remains constrained by current limitations in multistep reasoning accuracy. Error compounding across long agentic chains, coupled with challenges in uncertainty quantification and task-specific calibration, hinders their application in high-stakes regulatory contexts. First proof-of-concept demonstrations, however, without peer-review, exist [38], which introduces TxAgent, an AI agent designed for therapeutic reasoning that leverages multi-step reasoning and real-time biomedical knowledge retrieval across a toolbox of 211 tools. However, robust governance, validation frameworks, and error-mitigation strategies must be developed before agentic AI can become a mainstay in regulatory toxicology [7]. A critical caveat concerns hallucination—the generation of plausible but false outputs. Mitigation strategies include retrieval-augmented generation (RAG), ensemble verification, and mandatory human-in-the-loop review before high-impact conclusions are adopted. Continuous benchmark testing for factual accuracy should accompany every agentic deployment. Very soon, we will see agents capable of integrating and manipulating a wide range of tools—constantly running, dynamically scaling, and even cloning themselves in response to new signals. For toxicology, this could manifest as a continuous monitoring agent across the human exposome, capable of both interpreting exposure signals and initiating mitigation or hazard assessment actions. These agentic models will not just analyze data, but self-improve by interacting with real-world testing environments. Their value will hinge on access to experiments, not just pretraining. The ability to design, execute, and learn from minimally harmful yet maximally informative experiments will become a defining competitive advantage, especially compared to static models trained on legacy datasets. This ties directly to our points around bias, causality, and the epistemic limits of black-box AI. Experimental feedback loops can shift AI from correlation to true scientific inquiry. Latest development include Recursive Self-improving AI (RSI),^27^ i.e., systems capable of autonomously enhancing their own intelligence, algorithms, and architecture through iterative self-modification cycles. Unlike traditional AI that requires human intervention for improvements, RSI systems improve their ability to self-improve, creating a feedback loop that can lead to exponential growth in intelligence and capabilities; their use is currently mostly limited to coding and solving mathematical problems, but they show a direction where AI independently finds problem solving strategies.

*Digital twins*—computational replicas of biological systems informed by individual data—are emerging as powerful platforms for personalized and predictive toxicology. In combination with AI, digital twins can simulate chemical exposure scenarios, dynamically model organ-specific responses, and support patient-centric safety decisions. In toxicology, virtual twin systems are being developed using integrated PBPK (physiologically based pharmacokinetic) models, exposure datasets, and ~ omics signatures to predict real-time responses under different conditions. These models allow for scenario testing, dose extrapolation, and early identification of susceptible subpopulations. When implemented with cloud-based infrastructures, digital twins can also be embedded in decision-support systems for regulators or industry risk managers. The ONTOX project^28^ [39, 40] and NICEATM's Integrated Chemical Environment (ICE)^29^ already use components of this paradigm, positioning it as a next-generation NAM for regulatory toxicology [32]. A broader EU Virtual Human Twins (VHT) Initiative^30^ launched in 2023 to integrate multi-scale computational models of human physiology. It combines the EDITH^31^ (Ecosystem for Digital Twins in Healthcare) roadmap for ecosystem development led by the Virtual Physiological Human Institute (VPHi)^32^ and Horizon Europe funding (€80 million) for research on patient-specific disease models and a €24 million digital platform (Digital Europe Programme) for model integration and validation. The Initiative completed its first demonstration phase in mid-2025, validating cardiac and hepatic digital-twin modules within the EDITH WP2 framework. These milestones confirm that AI-driven virtual humans are moving from concept to early implementation in regulatory research.

*Generative AI*, particularly transformer-based architectures such as generative adversarial networks (GANs) and large language models (LLMs), is reshaping toxicological workflows [14, 41]. These models are capable of generating synthetic chemical structures, filling in missing exposure data, simulating literature summaries, and even producing mechanistic hypotheses. For example, GANs have been used to create synthetic toxicogenomic profiles that mirror real data distributions, providing additional training material for rare endpoints. LLMs can automate literature triage, protocol drafting, and annotation of legacy studies, thereby accelerating evidence synthesis. However, generative models must be carefully validated to prevent the propagation of artifacts or hallucinated outputs, especially when informing regulatory decisions. Nevertheless, their creative and data-augmentation capabilities make them powerful allies in addressing the data scarcity, heterogeneity, and synthesis [42] challenges endemic to toxicology [43]. Not all data types should be synthetically reproduced. Generation of identifiable human exposure records, proprietary in-vivo datasets, or clinical information without governance approval poses ethical and legal risks. Generative pipelines must therefore include safeguards and provenance tagging to prevent inadvertent re-creation of protected data.

*Neuromorphic and Quantum Computing*—Emerging hardware paradigms such as neuromorphic computing and quantum computing offer long-term prospects for expanding the scope of AI in toxicology by increasing computational power. Neuromorphic chips mimic the architecture of biological neural systems, allowing energy-efficient processing of complex, temporal data such as electrophysiology or toxicokinetics. These systems could enable real-time learning from biosensors or in vitro time-course data, supporting dynamic toxicity assessment. Quantum computing, although still nascent, holds the potential to revolutionize molecular simulations, multi-objective optimization, and network-based causal inference—tasks that are computationally prohibitive for classical systems. For example, simulating toxicant interactions with protein networks or predicting emergent properties in chemical mixtures could benefit from the exponential speedup quantum algorithms promise. While practical deployment is years away, early investment in quantum-aware toxicology models and neuromorphic architecture optimization will prepare the field for next-generation breakthroughs.

### Ranking Ethical Priorities for AI in Toxicology

We have recently described two visions for the future of AI-facilitated science, i.e., first a more and more AI-driven scAInce [44] and the development of toxicology toward a Human Exposome Project [45] through AI [46] with considerable ethical challenges [47]. To translate ethical discourse into action, we introduce a ranked priority matrix (Table 1) classifying immediacy of ethical concerns: short-term—bias and transparency; mid-term—consent, data sovereignty, and workforce impact; long-term—algorithmic autonomy and sustainability. This ranked matrix translates abstract ethical discourse into staged action priorities, providing regulators and researchers with a pragmatic roadmap for responsible AI integration in toxicology. This framing provides a practical roadmap for regulators and researchers.

**Table 1:** Ranked ethical priorities for AI in toxicology

Time Horizon	Ethical Focus Area	Core Challenge	Operational Imperative / Example Implementation
Short-term (0–3 years)	Bias and Fairness	Historical toxicology data reflect systemic, methodological, or demographic biases that can propagate through AI models	Conduct *bias audits* across datasets and algorithms; employ fairness metrics (e.g., demographic parity); establish independent oversight panels
	Transparency and Explainability	Deep models act as “black boxes,” eroding trust and regulatory acceptance	Mandate model documentation (model cards); adopt explainable-AI toolkits (e.g., SHAP, LIME); publish interpretability benchmarks per OECD AI Principles
Mid-term (3–10 years)	Consent and Data Sovereignty	AI training often repurposes human or proprietary toxicology data without ongoing consent or governance	Embed *dynamic consent* frameworks; align with GDPR/HIPAA; implement data-provenance tracking and CARE/FAIR compliance
	Workforce Impact and Accountability	Automation shifts skill demands, risking displacement or deskilling of laboratory and regulatory personnel	Invest in *up-skilling* programs for AI literacy; define shared human–machine accountability (“co-pilot” model)
Long-term (10 + years)	Algorithmic Autonomy	Agentic or self-improving AI may operate beyond human interpretability or oversight	Require *human-in-the-loop* governance; implement continuous audit trails, sandboxed deployment, and ethical “kill switches.”
	Sustainability and Societal Equity	Compute intensity and unequal access threaten environmental and global fairness goals	Promote *green AI* metrics (energy transparency, carbon reporting); ensure equitable access for low-resource regulators through federated infrastructures

### Timelines

To contextualize development timelines, near-term (2025–2030) adoption is expected for federated and xAI workflows; medium-term (2030–2035) for agentic and digital-twin integration; and long-term (> 2035) for neuromorphic and quantum computing applications. These projections provide a temporal roadmap for research and regulatory preparation.

## From Validation Bottlenecks to E-Validation and Companion Agents

The transformative potential of AI-based NAMs in toxicology is increasingly recognized, but it remains bottlenecked by legacy validation frameworks that were originally designed for static, animal-centric assays. Traditional validation—rooted in ring trials, protocol freezing, and one-to-one concordance with animal models—cannot accommodate the dynamism, complexity, and continuous learning capabilities of modern AI systems [7]. Similar validation bottlenecks are recognized in other domains such as radiology, genomics, and medical device software. The FDA’s continuous-learning framework and EMA’s adaptive evidence models offer instructive precedents for how performance-centric validation can coexist with regulatory rigor [38, 48]. The urgent need for faster, more adaptive, and scientifically robust validation pathways has led to the emergence of the e-validation framework, which reimagines the validation process through the lens of AI, translational science, and mechanistic relevance. At the heart of e-validation are five interlocking AI-powered components [5]:

1. *Smart Reference Chemical Selection* Instead of relying on historical reference compounds chosen by expert consensus—which often results in overused, biased, or mechanistically narrow test sets—e-validation deploys clustering algorithms (e.g., k-means, Density-Based Spatial Clustering of Applications with Noise (DBSCAN)) [49] to ensure structurally and mechanistically diverse chemical spaces are covered. Reference chemicals are selected not only for their availability and historical data but also for their relevance to human biology and the mechanistic diversity they represent. Chemical clustering leverages molecular fingerprints, bioactivity profiles, and AOP ontology embeddings to ensure mechanistic and structural diversity. This increases coverage of chemical space and improves transferability of validation results across compound classes. These selections can be iteratively refined as new data emerges, and integrated with public databases to ensure regulatory applicability.
2. *Simulation of Validation Study Outcomes* Validation no longer needs to be confined to wet-lab trials with fixed endpoints. The e-validation framework [50] uses virtual validation studies that combine physiologically based pharmacokinetic (PBPK) and in vitro biokinetic modeling with QSAR predictions, systems biology networks, and statistical optimization to pre-test study designs [51–53]. By simulating experimental conditions—such as dosing ranges, time points, and replication schemes—AI-driven modeling enables scenario testing, power calculations, and sensitivity analyses before laboratory execution. This allows identification of designs with the highest informational yield and reproducibility, while minimizing resource demands and ethical costs.
  Simulated validation thus becomes an evidence-preparation stage that anticipates real-world outcomes, identifies key sources of uncertainty, and guides optimal experimental design [32, 50, 54]. Iterative simulations can compare study architectures across virtual populations, generating quantitative predictions of expected variance and robustness. The result is not a replacement for empirical testing but a refinement step that makes subsequent physical validation faster, more reproducible, and better aligned with human-relevant biology.
3. *Mechanistic Cross-Validation *via* Literature Mining* Mechanistic validation [55, 56], often under-emphasized in traditional frameworks, becomes a cornerstone of e-validation. AI—particularly large language models (LLMs) and graph neural networks—can mine the literature for evidence of pathway activation, AOP concordance, and causal inference [28]. Using tools like NLP-enhanced Bradford Hill assessments [28], mechanistic cross-validation aligns NAMs with human-relevant biological processes rather than simply mimicking animal results.
4. *AI-Enhanced Training Dashboards* A core component of the validation process is ensuring consistent implementation across participating labs. AI-enhanced dashboards offer protocol customization, troubleshooting, video support, and live guidance for end users. This fosters consistency and transparency across validation studies while democratizing access to best practices. Post-validation, these dashboards serve as knowledge transfer tools for regulatory and industry uptake. Current exemplars include the OECD AI Platform^33^ and NIH NAM-Navigator^34^ platforms, which provide standardized dashboard hosting under neutral, multi-stakeholder governance. Such public–private stewardship minimizes conflicts of interest while ensuring transparent, auditable dissemination of training materials.
5. *Continuous Performance Monitoring and Companion Agents* The concept of companion post-validation agents represents an evolution in how validated methods are maintained over time. These autonomous AI systems monitor performance metrics in real-world use, perform back-testing as new data accumulates, and flag when retraining or re-validation may be necessary. This marks a departure from the current static view of validation and introduces a lifecycle model where AI-based NAMs evolve alongside evidence and regulatory requirements [20].

This entire e-validation framework resonates with and operationalizes the TREAT criteria—Trustworthiness, Reproducibility, Explainability, Applicability, and Transparency—proposed for AI applications in regulatory contexts [6]. Importantly, e-validation complements the modular and fit-for-purpose concepts already gaining traction in validation science. It also aligns with the translational medicine paradigm, particularly through the use of qualified biomarkers to benchmark predictive and mechanistic validity, not merely historical concordance with animal models [32].

On a philosophical level, e-validation addresses key ethical and epistemological critiques of the existing validation paradigm. Validation, as discussed [38], must shift from being a static gatekeeper to a dynamic enabler—one that ensures scientific credibility without stifling innovation [33]. It must accommodate uncertainty, embrace adaptive designs, and prioritize human health relevance over legacy comparators.

**Fig. 3:**
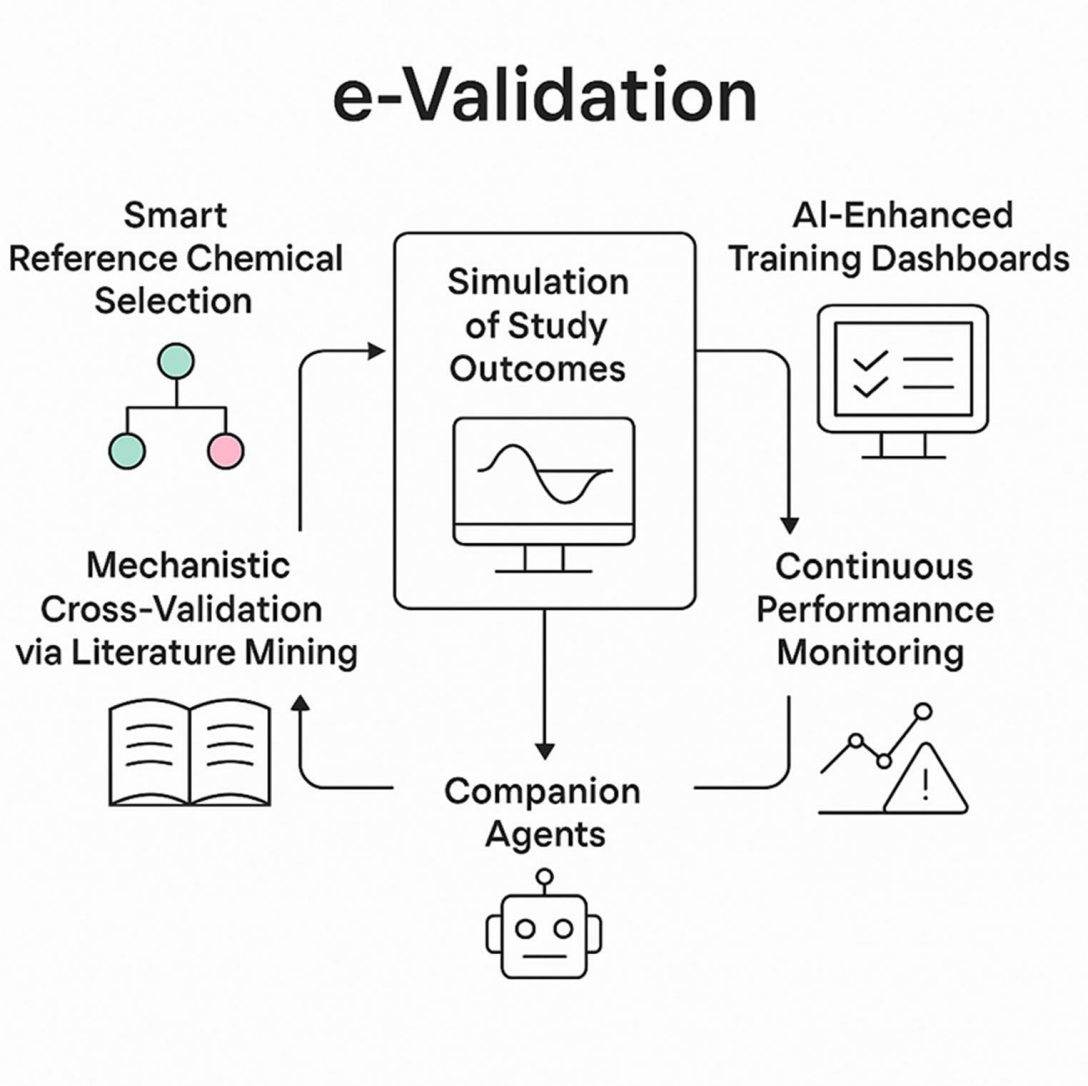
**The concept of e-validation**. The figure depicts the key components of an AI-facilitated validation process for in vitro, computational and AI-based methods [7, 51]

In sum, e-validation and companion agents together propose a paradigm shift from "validate and forget" to "validate, monitor, and evolve." This approach offers not only a scientific upgrade but also an ethical one—aligning toxicology with the values of animal replacement, human relevance, and continuous improvement [34]. Importantly, AI need not imply immediate full replacement of animal studies. Progressive refinement and reduction through AI-enabled prioritization can drastically decrease animal use while maintaining data continuity. The e-validation framework thus supports all 3Rs—replacement, reduction, and refinement—within a unified strategy (Fig. 3).

### Bridging Innovation and Oversight

The surge in AI capabilities for toxicology—spanning predictive modeling, data harmonization, mechanistic insight generation, and in silico experimentation—has transformed what is technically possible. However, this innovation outpaces existing validation, governance, and oversight structures, prompting a critical reckoning within regulatory science. Bridging this innovation-oversight divide is essential to harness AI's potential responsibly, credibly, and equitably. This section explores three central dimensions: trust and epistemology, governance frameworks, and the ethical–political context of regulatory AI [35].

### Rethinking Trust in the Age of Algorithmic Prediction

*AI's epistemological disruption* to toxicology centers on the tension between accuracy and understanding. Classical validation frameworks emphasize reproducibility, transparency, and mechanistic clarity. Yet many AI systems, particularly deep neural networks, offer robust performance without interpretable logic or consistent reproducibility, particularly when outputs depend on random initializations or iterative retraining [36].

This raises profound questions:

- Can we trust AI systems we do not fully understand?
- Should we prioritize empirical performance over mechanistic transparency?
- What constitutes sufficient evidence for regulatory confidence?

While some argue that non-reproducible models cannot meet the standards of toxicology—a field long reliant on Good Laboratory Practice (GLP) and standard operating procedures—others propose a performance-centric validation, where a model is trusted if it consistently delivers accurate predictions under predefined conditions [37]. This tension recalls debates in medicine over "black-box" diagnostics that outperform clinicians but resist human explanation.

One potential resolution is layered trust architecture, where models are conditionally accepted for tightly scoped use-cases (e.g., screening or prioritization) but require additional validation layers—such as explainable AI (xAI), post-deployment monitoring, and uncertainty quantification—for broader applications [38]. This reminds of the “*incremental validation*” we suggested earlier expanding applicability domains continuously [57].

#### Building Adaptive, Collaborative Oversight Frameworks

To integrate AI safely into toxicology, oversight must evolve from static validation to adaptive lifecycle governance. This shift is already underway in adjacent sectors. The FDA's action plan for AI/ML-based software^35^ emphasizes the continuous learning nature of these models and proposes pre-determined change control protocols, real-time monitoring, and transparency measures [39]. Similarly, OECD and EU initiatives now support modular, fit-for-purpose validation and the use of evidence-weighted frameworks that include biomarker performance, mechanistic validity, and confidence intervals, rather than binary concordance with animal models [40]. These initiatives are advancing steadily: the OECD modular validation pilot completed two inter-laboratory case studies in 2025, and the EU ONTOX project, pioneering a lot of AI use in toxicology, reached mid-term milestones including AOP alignment and a publicly released model catalogue.

A centerpiece of this new oversight landscape is the e-validation framework (see above). This approach aligns with the TREAT principles—Trustworthiness, Reproducibility, Explainability, Applicability, and Transparency—which form an emerging gold standard for AI in safety–critical contexts. Rather than validating a model once and freezing it, the emphasis shifts to dynamic credibility, where validation is an ongoing, evidence-responsive process [6].

Global harmonization is also critical. Collaborative initiatives such as the Global Coalition for Regulatory Science Research (GCRSR)^36^ and International Cooperation on Alternative Test Methods (ICATM)^37^ aim to standardize AI validation expectations across borders, avoiding the emergence of divergent regulatory ecosystems that could fragment trust or stall progress.

### Ethics, Equity, and the Politics of Risk

While the technical capabilities of AI-enabled NAMs continue to expand—delivering increasingly accurate, scalable, and human-relevant predictions—regulatory science must grapple with deep, foundational questions that challenge long-held assumptions about evidence, trust, and oversight [58]. One such question concerns the reliability of non-reproducible but consistently accurate AI models. Traditionally, reproducibility has been a cornerstone of toxicological validation, but AI models—especially those employing deep learning or probabilistic outputs—often operate with degrees of stochasticity that defy exact duplication. This calls for a paradigm shift in how reproducibility is defined and evaluated, especially if models demonstrate performance stability over time and across datasets [7].

Another core issue is the requirement for explainability. Should the ability to interpret an AI model's decision pathway be a precondition for regulatory acceptance, or is empirical accuracy alone sufficient in specific contexts? While many support explainability as a matter of transparency and trust, Bhuller et al. remind us that ethical principles such as autonomy and open decision-making also demand interpretability, especially in decisions that affect public health or environmental protection [50]. Ensuring that stakeholders can make informed choices about risk assessments aligns with both scientific integrity and moral norms around fairness and accountability.

Equally pressing is the question of how to manage and correct for the biases embedded in legacy toxicological datasets, many of which were generated using outdated or ethically questionable practices. These datasets—often used to train AI models—carry forward implicit assumptions and structural disparities. Without active measures to audit and mitigate these biases, AI may amplify, rather than eliminate, existing inequities in risk assessment. Ethical principles such as "reduce disparities" and "maintain respect and trust," as articulated in Bhuller et al.'s projector model, provide important guidance for navigating these challenges [50].

In response to these dilemmas, consensus is emerging around a "co-pilot" model for AI integration into regulatory workflows. Rather than replacing human expertise, AI is envisioned as an analytical assistant: augmenting human reasoning with speed, scope, and statistical rigor, while keeping the final decision authority with human risk assessors. This reflects an ethic of adaptability and shared decision-making, balancing innovation with oversight in a way that aligns with both public trust and institutional responsibility.

Moreover, bridging innovation and oversight requires active participation in global harmonization initiatives. Regulatory bodies such as the U.S. FDA are setting new precedents with their lifecycle-based principles for AI/ML models, emphasizing continual model monitoring, retraining protocols, and transparency obligations [59]. Meanwhile, the OECD and its members have adopted flexible, tiered validation strategies that accommodate emerging technologies and uphold principles like "fit-for-purpose" and "evidence-based decision-making." Global platforms such as the GCRSR promote capacity-building through training and joint guidance efforts, ensuring that both high- and low-resource countries can participate in and benefit from the AI transformation of toxicology.

These developments represent not only a modernization of regulatory science, but also a reassertion of its ethical mandate. As emphasized in the projector model proposed by Bhuller et al., ethical principles such as openness, stakeholder engagement, fairness, and the One Health perspective must be integral to every phase of risk decision-making—from problem formulation and data generation to model deployment and post-validation monitoring [50]. In this light, oversight is not an obstacle to innovation, but its ethical scaffold—ensuring that new technologies serve not only efficiency and accuracy but also justice, transparency, and the collective well-being of humans, animals, and the environment.

To navigate these tensions, ethical AI for toxicology must embed:

- *Bias audits and data provenance tracking*, to ensure inclusivity and accuracy across subpopulations,
- *xAI methods* to explain predictions in human-understandable terms,
- *Participatory governance*, involving civil society, regulators, scientists, and industry in model design, deployment, and oversight, and
- *Tiered access mechanisms*, ensuring that low-resource regulatory agencies can benefit from AI without ceding sovereignty to commercial or technical gatekeepers.

Bias audits are rapidly becoming an essential component of both traditional and AI-driven toxicological workflows, offering a structured way to identify and mitigate systematic errors that can distort study findings and mislead regulatory decisions. As discussed by Hartung et al. (2025), these audits focus on detecting various forms of bias—such as selection, performance, detection, attrition, and reporting biases—in experimental and computational studies [60]. In the context of AI-enabled toxicology, bias audits must expand beyond classical risk of bias tools to also interrogate data provenance, model assumptions, algorithmic fairness, and outcome reproducibility. This is critical given the susceptibility of AI systems to *data bias* (e.g., non-representative training sets), *algorithmic bias* (e.g., overfitting, feature selection bias), and *institutional bias* (e.g., confirmation bias embedded during model development). As such, effective bias audits should integrate human-led domain expertise with automated tools for bias detection—such as SHAP or LIME for model explainability, or AI-enabled protocol checkers for methodological consistency. The goal is not only to flag risks, but to estimate the *probabilistic impact and direction* of those biases on predicted outcomes, ultimately guiding safer, more equitable chemical assessments.

Moreover, as discussed already, a co-pilot paradigm is gaining favor, where AI may offer expansive data integration, mechanistic modeling, and scenario simulation, but final decisions remain with expert risk assessors who can weigh context, uncertainty, and ethical nuance. This co-pilot model provides a pragmatic equilibrium between innovation and oversight, autonomy and control, speed and trust.^38^

In conclusion, bridging innovation and oversight requires nothing less than a rethinking of what validation means in the age of AI. Static frameworks must give way to dynamic ecosystems. Reproducibility must be complemented by robustness. Trust must be earned through transparency, feedback, and performance. Only then can toxicology fully benefit from the predictive power of AI while remaining true to its ethical commitments and societal mandate [6, 61].

## AI Readiness Landscape: From Capacity to Consequence

The evolving landscape of AI readiness—spanning technological capacity, cost, trust, and institutional adoption—signals a pivotal moment for its integration into toxicology. The *2025 Stanford AI Index Report* [62] provides a comprehensive, global benchmark of this transformation, with findings that directly reinforce the feasibility and urgency of embedding AI into toxicological science and regulatory decision-making.

### Scaling with Affordability and Accessibility

One of the most striking developments is the dramatic drop in inference cost for large language models. A system performing at the level of GPT-3.5 now costs less than $0.07 per million tokens, down from $20 in late 2022—a > 280-fold reduction in 18 months [54]. This cost democratization, coupled with the rise of small, efficient models (e.g., Microsoft's Phi-3-mini with 3.8B parameters), expands access for academic labs, startups, and public health agencies alike.

Meanwhile, model performance is saturating at the frontier: the Elo score gap between the top and 10th-ranked models has narrowed from 11.9% to just 5.4% in one year, and the top two models are now separated by only 0.7%. This convergence marks a transition from raw power to domain specialization, interpretability, and real-world integration—a shift that favors toxicology applications where explainability and regulatory trust are paramount [54].

### Institutionalization of AI in Biomedicine

AI is increasingly embedded in real-world health technologies. In 2023 alone, the FDA approved 223 AI-enabled medical devices, up from just 6 in 2015 [54]. Simultaneously, domain-specific foundation models like Med-Gemini,^39^ ChexAgent,^40^ and EchoCLIP [63] are redefining how AI processes clinical, radiological, and ~ omics data.

These developments parallel trends in toxicology, where in silico predictions, high-content screening, and organoid data require robust interpretive frameworks. The normalization of AI across regulatory medicine validates the prospect of similar uptake in chemical risk assessment—particularly for evidence-weighted read-across, developmental neurotoxicity, and adverse outcome pathway (AOP) modeling [7].

### Trust Gaps and the Rise of Responsible AI Benchmarks

Despite technical advances, responsible AI (RAI) practice lags behind deployment. The number of reported AI incidents increased by over 56% in 2024 [54], while only a fraction of models are evaluated on emerging safety and transparency benchmarks like HELM Safety^41^ [64] or Fact-Checking Transparency Benchmarks (FACTS).^42^ Noteworthy, AIR-Bench 2024^43^ is a benchmark based on risk categories from regulations and policies evaluates models on a comprehensive taxonomy of AI risks (currently 8 government regulations and 16 company policies into a four-tiered safety taxonomy with 314 granular risk categories) enabling standardized evaluation of AI model safety across jurisdictions and regulatory frameworks. They provide standardized benchmarks, open-source code, and public leaderboards to ensure transparency in evaluating AI models, especially Large Language Models, including aspects like safety, bias, and toxicity (N.B., “toxicity” in the context of AI models refers to the generation or amplification of harmful, offensive, or malicious content by an artificial intelligence system).

We will have to explore how this translates to possible benchmarks for AI in the safety sciences and it underscores the relevance of the proposed TREAT framework (Trustworthiness, Reproducibility, Explainability, Applicability, Transparency) [6]. The AI Index data supports the notion that post-validation companion agents and continuous uncertainty monitoring are not aspirational concepts—but necessary guardrails for high-stakes applications [7].

### Strategic Implications for Toxicology

The convergence of accelerated compute capabilities, diminishing inference costs, plateauing performance across frontier models, and the mainstreaming of regulatory frameworks for AI has created fertile ground for the transformation of toxicology. What once appeared speculative—AI-enabled predictive toxicology replacing traditional animal-based paradigms—is now increasingly feasible. These developments reflect not just a shift in technology, but a profound maturation in the infrastructure and collective mindset surrounding AI in the life sciences. As such, the field stands at a pivotal moment: the readiness for large-scale adoption of AI-based toxicology is no longer a question of possibility, but of strategy. It is now more about trust building and change management than about technological development.

To seize this momentum, coordinated action is essential across several key fronts. First, the development and deployment of benchmarking frameworks tailored to toxicological applications is crucial. Tools like HELM and AIR-Bench, originally developed for broader AI model safety evaluations, must be adapted to encompass toxicology-specific endpoints, such as genotoxicity, developmental neurotoxicity, or organ-specific adverse effects. Such benchmarks will provide standardized, comparative performance metrics and foster trust in AI-based predictions across regulatory and research domains.

Second, model auditability must become a cornerstone of AI deployment in toxicology. Integrating explainable AI (xAI) techniques—such as SHAP values, saliency maps, i.e., visual or numerical representations that highlight the most important regions of an input that significantly influence a machine learning model’s prediction, or feature attribution tools, i.e., methods and software that help interpret and explain the predictions of complex machine learning models by quantifying the contribution of each input feature to a given prediction,—will help regulators, scientists, and other stakeholders interpret predictions and trace them back to specific data inputs or mechanistic reasoning. This integration is not merely a technical enhancement; it is an accountability mechanism that ensures AI models remain interpretable, responsive to feedback, and transparent in their assumptions and outputs.

Third, achieving the full promise of AI-based toxicology requires robust global data-sharing frameworks. Given the distributed nature of toxicological data—ranging from industry submissions to academic omics studies and regulatory exposure registries—traditional centralized databases are no longer sufficient. Federated learning and encrypted collaboration platforms offer a way forward, enabling decentralized toxicology consortia to jointly train models without compromising proprietary or sensitive information. This is particularly promising for applications like chemical similarity learning, read-across modeling, and transfer learning for rare or novel endpoints. Increasingly, experience is gained from large pharma consortia on toxicology data sharing and analysis such as eTox,^44^ eTRANSAFE^45^ [65] and VICT3R^46^ [66] by the Innovative Medicines Initiative (IMI), now Innovative Health Initiative (IHI). eTox created a large, shared toxicology database (eTOXsys) containing information from over 8,000 studies on nearly 2,000 compounds. eTRANSAFE integrated over 10,000 pharmacological studies from pharmaceutical companies into a unified system, called ToxHub. VICT3R is a public–private partnership launched in 2024 dedicated to reducing animal use in toxicology research by developing Virtual Control Groups (VCGs) based on data sharing by 23 pharmaceutical companies and an increasing number of Contract Research Organizations (CRO). It was developed out of a workshop organized by our center [67]. These projects set a new standard for data sharing in pharmaceutical safety by building a secure, standardized, and collaborative data ecosystem that will continue to benefit drug development and regulatory science. Nonetheless, federated learning complicates standardization and auditability. Harmonized metadata ontologies, cryptographically signed model updates, and inter-node proficiency testing are needed to ensure quality control and consistent ethical compliance across decentralized infrastructures.

Finally, no discussion of strategy is complete without addressing ethical oversight. As AI systems become embedded in regulatory and public health decisions, the inclusion of equity audits in the design and deployment of new approach methodologies (NAMs) is essential. These audits should examine not only the technical performance of AI models across demographic subgroups, but also the representativeness of training datasets, the inclusiveness of development processes, and the potential for disproportionate impacts on vulnerable populations. Ethical governance must be integrated from the outset—not as an afterthought, but as a core design principle.

Together, these actions form a blueprint for scaling AI in toxicology both responsibly and effectively. They recognize that readiness is not only about technological capability, but about cultural, regulatory, and ethical alignment. As the field advances, these pillars will shape the transition from fragmented innovation to a harmonized, global infrastructure for human-relevant safety science.

## Outlook: From Disruption to Reinvention

Artificial intelligence is poised not merely to streamline existing toxicological methods but to fundamentally transform how we approach chemical safety assessment. In the regulatory context, AI-based NAMs are expected to integrate directly into the pre-clinical–to–clinical continuum. Digital-twin and probabilistic simulation models can inform micro-dosing, adaptive trial design, and post-market pharmacovigilance, bridging current gaps between toxicology and clinical risk evaluation. Rather than replicating the outcomes of traditional animal studies, the true potential of AI lies in generating human-relevant, mechanistically informed predictions that are more accurate, ethical, and actionable. This shift represents a reinvention of toxicology itself, aligning the field with the imperatives of precision public health, sustainable innovation, and evidence-based policy.

Realizing this vision requires a comprehensive reevaluation of the data that drive toxicological inference. It begins with a decisive commitment to improving data quality—not only in terms of statistical robustness or completeness, but also in how representative datasets are of human biology, diverse populations, and realistic exposure scenarios. Poorly curated or biased training data can entrench historical errors, particularly when drawn from outdated or poorly standardized animal studies. Therefore, the toxicology community must invest in systematic data FAIRification, curated repositories, and the inclusion of underrepresented endpoints and vulnerable subpopulations.

Parallel to this, the field must advance xAI and causal modeling frameworks that move beyond correlational outputs toward biological intelligibility. Unlike traditional black-box models, xAI tools can identify the mechanistic underpinnings of predicted effects, providing transparency that fosters both regulatory trust and scientific insight. Causal inference frameworks—such as counterfactual reasoning, graphical models, and pathway reconstruction—enable researchers to trace predicted outcomes back to molecular initiating events or key events in adverse outcome pathways. This mechanistic clarity is not only scientifically rigorous, but also essential for replacing animal models with human-relevant, ethically grounded alternatives.

Equally transformative is the need to embrace validation paradigms that are dynamic, iterative, and performance-centered. The e-validation framework exemplifies this shift by integrating AI tools for reference compound selection, simulation-based protocol design, and post-validation surveillance. Rather than freezing test methods at a single point in time, adaptive validation enables continuous learning, uncertainty monitoring, and context-specific calibration. This is particularly vital for AI models, which evolve with incoming data and may respond to environmental, demographic, or regulatory changes in real-time.

Ultimately, the reinvention of toxicology through AI demands a reimagining of regulatory science itself—not as a rigid gatekeeper of past standards, but as a co-evolving, evidence-driven system capable of learning and adapting alongside the tools it evaluates. Regulatory frameworks must shift from static validation checklists to lifecycle governance models, incorporating probabilistic risk estimates, digital trust audits, and stakeholder-centered transparency protocols. As AI systems increasingly power both prediction and decision-support, the role of regulators will expand to include oversight of algorithmic integrity, post-market surveillance, and equitable deployment.

This reinvention is not merely a technological upgrade—it is a paradigm shift. It offers an opportunity to align toxicology with 21st-century values: replacing harm with prevention, opacity with transparency, and extrapolation with human specificity. In doing so, AI can fulfill its promise not only to accelerate toxicological science, but to transform it into a more just, responsive, and human-centered discipline.

## Conclusions

Artificial intelligence is no longer a distant promise for toxicology—it is the transformative force now shaping the discipline's future. Over the course of this review, we have mapped the landscape of AI-driven change in toxicology across technical, regulatory, ethical, and strategic dimensions. These developments mark a critical inflection point: toxicology is transitioning from adapting AI to co-evolving with it, entering a new era of mechanistically grounded, human-relevant, and dynamically validated safety science.

Central to this transformation is the recognition that AI does not merely automate legacy workflows—it reinvents them. Multimodal AI, causal inference, generative modeling, and physics-informed learning have expanded the predictive and interpretive capabilities of toxicology beyond what animal models can achieve. Combined with real-time data access, edge computing, and federated learning, these tools have made toxicology more responsive, personalized, and ethically aligned. The emergence of digital twins and agentic AI systems further signals the rise of intelligent, autonomous toxicology pipelines, capable of simulation, prediction, and adaptation at unprecedented scale.

However, the realization of AI's full potential demands more than technical readiness—it requires a transformation in how toxicology conceptualizes validation, governance, and trus**t**. The e-validation framework, grounded in the TREAT principles, exemplifies this shift [6]. It introduces adaptive, AI-powered modules for selecting reference chemicals, simulating outcomes, cross-validating mechanisms, and ensuring lifecycle monitoring through post-validation companion agents [7].

At the same time, the ethical landscape must evolve alongside the technical. Bias audits, participatory governance, and equity-aware development practices are no longer optional—they are foundational. As AI systems increasingly shape regulatory decisions, these systems must be interrogated for their fairness, representativeness, and inclusivity. The co-pilot model—where AI augments rather than replaces human judgment—offers a pragmatic path forward, one that combines the scale and speed of algorithms with the contextual, moral, and experiential insights of human decision-makers.

The global readiness for AI in toxicology is accelerating. With inference costs plummeting, model performance stabilizing, and regulatory precedent expanding—most notably through the FDA's endorsement of AI-enabled diagnostics—the infrastructure is now in place. The challenge ahead lies in coordination: establishing shared benchmarks, harmonized validation frameworks, secure data-sharing ecosystems, and inclusive ethical oversight. These are not merely supporting structures; they are the scaffolds upon which the future of responsible AI-driven toxicology will be built.

In sum, this moment offers more than an opportunity for innovation—it demands reinvention. AI offers the means not just to do toxicology faster or cheaper, but to do it better: with more precision, with greater relevance to human biology, and with deeper ethical integrity. It challenges the field to move beyond inherited paradigms of animal testing and binary endpoints, toward a future where chemical safety science is data-rich, mechanistically informed, and globally inclusive.

If this vision is realized—through science, governance, and trust—AI will not merely assist toxicology. It will elevate it. And in doing so, it will help build a future where the assessment of chemical risk is not only smarter, but safer, more equitable, and fundamentally more humane.

## Key References

- Hartung T, Kleinstreuer NC. Challenges and opportunities for validation of AI-based new approach methods. ALTEX 2025;42(1):3-21.
  - ○ Conceptual paper on validation of AI-facilitated methods coauthored with the then head of the US validation body.
- Hartung T, Whelan, Tong W, Califf RM. Is Regulatory Science Ready for Artificial Intelligence? NPJ Digital Medicine 2025;8:200
  - ○ Opinion piece on the prospects of building trust into the regulatory use of AI coauthored with at the time FDA leadership and head of the EU validation body.
- Kleinstreuer N, Hartung T. Artificial Intelligence (AI) – it’s the end of the tox as we know it (and I feel fine)—AI for Predictive Toxicology. Arch Toxicol. 2024;98:735–754
  - ○ Extensive review of the history, current examples and future prospects of AI in toxicology coauthored with the then head of the US validation body.

## Data Availability

Data sharing is not applicable to this article as no datasets were generated or analyzed during the current study.

## Footnotes

1 https://comptox.epa.gov/dashboard/

2 https://qsartoolbox.org

3 https://ntp.niehs.nih.gov/whatwestudy/niceatm/comptox/ct-ice/ice

4 https://worldfair-project.eu/fair-implementation-profiles/

5 FDA. Artificial Intelligence and Machine Learning (AI/ML) Medical Devices. U.S. Food and Drug Administration Annual Report. 2024. Available at: https://www.fda.gov/medical-devices/software-medical-device-samd/artificial-intelligence-and-machine-learning-aiml-enabled-medical-devices (last accessed 25 Apr 2025).

6 https://crfm.stanford.edu/fmti/May-2024/index.html

7 Bommasani et al., 2024, https://crfm.stanford.edu/fmti/paper.pdf

8 https://xaitk.org

9 https://sites.research.google/med-palm/

10 https://spark.apache.org/docs/latest/index.html

11 https://www.ray.io

12 https://www.dask.org

13 https://www.geeksforgeeks.org/deep-learning/what-is-monte-carlo-mc-dropout/

14 https://docs.aws.amazon.com/prescriptive-guidance/latest/ml-quantifying-uncertainty/deep-ensembles.html

15 https://ermongroup.github.io/cs228-notes/inference/variational/

16 https://shap.readthedocs.io/en/latest/index.html

17 https://christophm.github.io/interpretable-ml-book/overview.html

18 https://cloud.google.com/vertex-ai

19 https://h2o.ai

20 https://www.automl.org/automl-for-x/tabular-data/auto-sklearn/

21 https://eur-lex.europa.eu/eli/reg/2016/679/oj

22 https://www.iso.org/standard/42001

23 https://www.oecd.org/en/topics/sub-issues/ai-principles.html

24 https://chain.link/education-hub/secure-multiparty-computation-mcp

25 https://eur-lex.europa.eu/eli/reg/2016/679/oj.

26 https://www.govinfo.gov/content/pkg/PLAW-104publ191/pdf/PLAW-104publ191.pdf

27 https://storage.googleapis.com/deepmind-media/DeepMind.com/Blog/alphaevolve-a-gemini-powered-coding-agent-for-designing-advanced-algorithms/AlphaEvolve.pdf

28 https://ontox-project.eu

29 https://ice.ntp.niehs.nih.gov

30 https://digital-strategy.ec.europa.eu/en/policies/virtual-human-twins

31 https://vito.be/en/projects/edith

32 https://www.vph-institute.org

33 https://oecd.ai/en/

34 https://oacu.oir.nih.gov/new-approach-methodologies

35 https://www.fda.gov/medical-devices/software-medical-device-samd/artificial-intelligence-and-machine-learning-software-medical-device

36 https://gcrsr.net

37 https://ntp.niehs.nih.gov/whatwestudy/niceatm/iccvam/international-partnerships/icatm

38 https://medium.com/data-science/top-10-data-ai-trends-for-2025-4ed785cafe16

39 https://research.google/blog/advancing-medical-ai-with-med-gemini/

40 https://stanford-aimi.github.io/chexagent.html

41 https://github.com/stanford-crfm/helm/blob/main/README.md

42 https://arxiv.org/html/2410.15135v2

43 https://arxiv.org/html/2407.17436v2

44 https://www.ihi.europa.eu/projects-results/project-factsheets/etox

45 https://etransafe.eu

46 https://www.vict3r.eu
